# The efficacy of short acquisition time using ^18^F-FDG total-body PET/CT for the identification of pediatric epileptic foci

**DOI:** 10.1186/s13550-024-01081-x

**Published:** 2024-02-26

**Authors:** Min Li, Xiao Cui, Huixin Yue, Chao Ma, Kun Li, Leiying Chai, Min Ge, Hui Li, Yee Ling Ng, Yun Zhou, Jianguo Shi, Yanhua Duan, Zhaoping Cheng

**Affiliations:** 1 Postgraduate Department, Shandong First Medical University, Shandong Academy of Medical Sciences), Jinan, China; 2 Department of Nuclear Medicine, The First Affiliated Hospital of Shandong First Medical University & Shandong Provincial Qianfoshan Hospital, Jinan, China; 3 Central Research Institute, United Imaging Healthcare Group Co., Ltd, Shanghai, China; 4 Department of Epilepsy Center, Children’s Hospital Affiliated to Shandong University, Jinan Children’s Hospital, Jinan, China

**Keywords:** Total-body PET/CT, Fast acquisition, Diagnostic performance, Epileptogenic zone, Pediatric

## Abstract

**Background:**

^18^F-FDG positron emission tomography (PET) plays a crucial part in the evaluation for pediatric epileptic patients prior to therapy. Short-term scanning holds significant importance, especially for pediatrics epileptic individuals who exhibited involuntary movements. The aim was to evaluate the effects of short acquisition time on image quality and lesion detectability in pediatric epileptic patients using total-body (TB) PET/CT. A total of 25 pediatric patients who underwent TB PET/CT using uEXPLORER scanner with an ^18^F-FDG administered dose of 3.7 MBq/kg and an acquisition time of 600 s were retrospectively enrolled. Short acquisition times (60 s, 150 and 300 s) were simulated by truncating PET data in list mode to reduce count density. Subjective image quality was scored on a 5-point scale. Regions of interest analysis of suspected epileptogenic zones (EZs), corresponding locations contralateral to EZs, and healthy cerebellar cortex were used to compare the semi-quantitative uptake indices of short-time images and then were compared with 600 s images. The comparison of EZs detectability based on time-dependent PET images was performed.

**Results:**

Our study demonstrated that a short acquisition time of 150 s is sufficient to maintain subjective image quality and lesion significance. Statistical analysis revealed no significant difference in subjective PET image quality between imaging at 300 s and 150 s (*P* > 0.05). The overall impression scores of image quality and lesion conspicuity in G60s were both greater than 3 (overall quality, 3.21 ± 0.46; lesion conspicuity, 4.08 ± 0.74). As acquisition time decreased, the changes of SUVmax and SD in the cerebellar cortex gradually increased (*P* < 0.01). There was no significant difference in asymmetry index (AI) difference between the groups and the AIs of EZs were > 15% in all groups. In 26 EZs of 25 patients, the lesion detection rate was still 100% when the time was reduced to 60 s.

**Conclusions:**

This study proposed that TB PET/CT acquisition time could be reduced to 60 s with acceptable lesion detectability. Furthermore, it was suggested that a 150 s acquisition time would be sufficient to achieve diagnostic performance and image quality for children with epilepsy.

## Background

Epilepsy is the second largest neurological disorder after stroke, affecting approximately 1% of the global population [1]. Epilepsy is also a common disease in children, with a prevalence of 0.5-1%, and its high disability rate and long course not only affect the quality of life of patients, but also lead to brain tissue damage and neuropsychiatric disorders, making it a worldwide public problem [2]. This disorder has a great impact on the growth and development, physiological and mental health of children [3], making the early detection of epileptogenic zone (EZ) in children highly crucial in order to accurately locate the epileptic foci, clarify the cause and type of epilepsy, and develop individualized and precise treatment plans for patients.

The diagnosis, evaluation, and treatment of epilepsy involve several imaging tools, including electroencephalography (EEG), CT, MRI, magnetic resonance spectroscopy, Magneto-encephalography (MEG), single-photon emission computed tomography (SPECT), and positron emission tomography (PET) [4]. Previous studies have revealed that PET is a crucial imaging technique for the preoperative assessment of epileptic foci in epileptic patients [5–8]. Traditional two-dimensional (2D) brain FDG scan acquisition needs 20 min per bed position and three-dimensional (3D) scan time needs 6 min per bed position [9], but total-body (TB) PET/CT scans total body in 10 min [10]. Compared to adults, children have difficulty controlling their behavior and sustaining a longer scanning time. A child’s ability to control their behavior and cooperate with the medical examination is dependent on their age and cognitive/emotional development [11]. Therefore, sedation is necessary for children who are difficult to cooperate with. Sedation in pediatric patients is a continuum and can result in respiratory depression, laryngeal spasm, impaired airway patency, apnea, loss of protective airway reflexes in patients, and cardiovascular instability [12–15]. If the short acquisition time scan can meet the diagnostic needs, the success rate of PET-CT scans can be greatly improved, and the potential harm to children caused by sedative drugs can be minimized. For patients suffering from claustrophobia, a short acquisition time can also significantly increase the likelihood of a successful scan.

The mass of PET imaging is closely related to the efficiency of detecting photon pairs emitted from positron-electron annihilation. The two main parameters that determine the efficiency of PET scanner detection are the length of the axial FOV and the sensitivity of the detector [16]. However, the sensitivity of the uEXPLORER PET/CT scanner with a 194-cm-long axial FOV can be increased by a factor of approximately 4–5 for brain imaging [17]. A newly-published study by Zhang et al. on short acquisition time TB PET/CT in 19 adult oncological patients concluded that TB PET imaging allowed acquisition time to be reduced down to 60- and 30-s while maintaining ^18^F-FDG kinetics and acceptable image contrast [18]. However, pediatric epilepsy patients need to be assessed separately due to the fact that children have a different distribution of weight and mass than adults. Additionally, epilepsy in the intermittent period has been revealed to show hypometabolism on ^18^F-FDG PET imaging, which is different from tumors, which show hypermetabolism.

In this study, we aimed to explore the effects of short acquisition time using uEXPLORER in the detection of EZs of PET images.

## Materials and methods

### Patients

This study retrospectively analyzed children with epilepsy who underwent craniocerebral PET-CT scanning, MRI and video EEG (VEEG) from October 2020 to May 2022. The inclusion criteria were as follows: (1) patients aged under 18 years old, and (2) surgical treatment was performed with pathological confirmation or (3) after the electrode tablet implantation, the symptoms resolved, and (4) patients underwent a PET examination at our hospital within two weeks before surgery or brain electrode tablet implantation. The exclusion criteria encompassed the following: (1) patients aged 18 years and older, (2) cases where epilepsy could not be definitively diagnosed through surgery or electrode tablet implantation with follow-up, and (3) instances where patients’ poor cooperation led to the occurrence of significant motion artifacts, thereby compromising the PET image quality. Informed consent was obtained from all patients’ legal guardians.

### Positioning

Standard brain imaging locations were used; the image plane is parallel to the canthus line. The movement of children should be avoided. Head fixation devices can be used to avoid movement of children.

### Imaging protocol

All patients were required to avoid vigorous exercise 24 h before the examination and fast for more than 6 h. All patients were injected with ^18^F-FDG (3.7MBq/kg) according to body weight. 600 s list mode PET data were collected on a 194-cm axial field panoramic PET-CT (uEXPLORER, United Imaging Healthcare, Shanghai, China). All 600 s data were used to reconstruct PET images, which were then divided into groups of 300, 150 and 60 s duration to simulate the rapid acquisition scene. For simplicity, these image series were referred to as G600, G300, G150, and G60 s in the rest of this paper. Ordered subset expectation maximization (OSEM) was used for the reconstruction of all PET images, with the following parameters: TOF and PSF modeling, 3 iterations and 20 subsets, matrix 192 × 192. Slice thickness 1.443 mm, FOV 300 mm (pixel size 3.125 × 3.125 × 1.443mm^3^), Gaussian post filter (3 mm), and all necessary corrections such as attenuation correction and scattering correction. All image evaluation was performed on a commercial medical image processing workstation (uWS-MI, United Imaging Healthcare).

### Analysis of PET/CT imaging

**Fig. 1:**
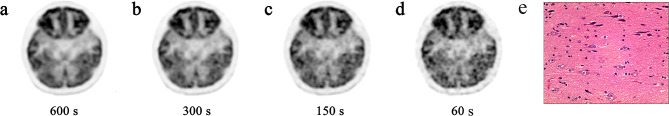
^18^F-FDG PET image of an 11-year-old female patient weighing 43 kg, who had been diagnosed with gangliocytoma by pathology, was reconstructed into brain axial views of 600, 300, 150 and 60 s (**a**–**d**). The overall image scores for the groups of 600, 300, 150, and 60 s were 5, 5, 4, and 3, respectively. (**e**) Histopathology finding of the patient, characterized by a mixed population of neoplastic glial cells and dysplastic neurons

Two nuclear radiologists, blinded to patient history and acquisition time, independently rated the subjective PET image quality using a 5-point Likert scale. The scale ranged from 1 (non-diagnostic) to 5 (excellent), with 1 being undiagnosable, 2 being considered inadequate diagnosis, 3 being considered acceptable for diagnosis, 4 being considered better than the average, and 5 being considered optimal. Examples of grades 5 − 3 (5: excellent, 3: fair) were illustrated in Fig. 1.

An experienced technician performed an objective image quality analysis under the supervision of a nuclear radiologist. Two 2-dimensional circular region-of-interests (ROIs) with an area of 100 mm² were drawn on a homogeneous area of the ipsilateral cerebellar cortex. The maximum standard uptake value (SUVmax) and standard deviation (SD) of ROI were recorded and average values were obtained to evaluate the image quality between different groups. The ROIs with an area of 100 mm² were drawn on the center area of epileptogenic zones and the corresponding contralateral regions, which were measured three times and the average value was calculated. To ensure the consistent size and location of the ROI in each patient image, the ROI of the epileptogenic regions and corresponding contralateral regions was first measured on G600 s and subsequently measured on G300, G150, and G60 s using the copy-paste function. The semi-quantitative uptake measurements of epileptogenic zones and corresponding contralateral locations were recorded: SUVmean of both EZ and contralateral. The Asymmetric index (AI) was used to locate and diagnose EZ, and the AI > 15% was regarded as abnormal [19]. AI is equal to the difference between the SUVmean of the EZ and the SUVmean of the contralateral divided by the sum of the two numbers multiplied by 200%. The corresponding locations of the micro-lesion identified on the PET images of G600 s with standard acquisition time were documented and served as a reference to estimate lesion detectability according to the EANM procedure guidelines for the duration of TB PET scan acquisition. Randomized orders of short acquisition time images were used to minimize recall bias. Images with excessive background noise or poor image quality were marked as undiagnosable lesions.

### Statistical analysis

Statistical analyses were performed using SPSS 26.0 software for Windows (IBM SPSS Inc., Armonk, NY, USA). The inter-rater agreement of the subjective score was evaluated using Fleiss kappa test. Kappa values of 0.41–0.60, 0.61–0.80, and 0.81-1.00 indicated moderate, substantial, and excellent agreement, respectively. For the subjective image quality analysis between different subsets, the Kruskal Wallis rank-sum test and Dunn’s post hoc test for multiple comparisons were used. To account for inter-patient differences in SUV, the Wilcoxon rank-sum test and Bonferroni correction were employed to compare objective image quality between different groups. SUVmax, SD and AI were calculated. Results were expressed as the differences of SUVmax, SD, and AI between G300 to G60 and G600s for each patient, and then the mean of these differences and SD differences were calculated for all patients. A *P*-value of < 0.05 was considered statistically significant.

## Results

### Patients

A total of 25 pediatric epileptic patients (10 males, 15 females) were enrolled. 23 patients underwent surgery and the symptoms resolved after the electrode tablet implantation in two individuals. The mean age of the patients was 5.28 ± 3.27 years, ranging from 1 to 14 years, and a mean body mass index (BMI) of 17.5 kg/m^2^, ranging from 13.9 kg/m2 to 28.3 kg/m^2^ (Table 1).

**Table 1:** Patient characteristics

	Mean ± SD	Range
Age (years)	5.28 ± 3.27	1–14
Weight (kg)	23.75 ± 15.33	8.5–80
Height (cm)	111.52 ± 24.80	73–168
Injected dose (MBq)	82.05 ± 41.70	36.63-237.54
Injected dose per weight (MBq/kg)	3.73 ± 0.80	2.95–6.09
Waiting time (min)	49.18 ± 9.90	36.53–68.04

### Image quality

There was significant agreement among raters regarding overall image quality, image noise and lesion conspicuity score (kappa = 0.895, 0.933, 0.867 respectively). The unified subjective image quality scores among G300, G150, and G60 s were then compared. The detailed results are shown in Table 2.

**Table 2:** Subjective image quality assessed by 5-point Likert scale

Image quality score	Group 300 s (range 1–5)	Group 150 s (range 1–5)	Group 60 s (range 1–5)	*P* value
Overall quality	4.79 ± 0.37	4.37 ± 0.55	3.21 ± 0.46	< 0.001
Image noise	4.67 ± 0.46	4.27 ± 0.52	2.73 ± 0.59	< 0.001
Lesion conspicuity	4.92 ± 0.27	4.62 ± 0.49	4.08 ± 0.74	< 0.001

All scores were presented as mean value ± SD and the images were reconstructed with a voxel size of 3.125 × 3.125 × 1.44 mm^3^

The G300 s showed excellent image quality, with all views rated between 4 and 5. The mean and SD of overall image quality, image noise and lesion conspicuity scores of G300 s were 4.79 ± 0.37, 4.67 ± 0.46 and 4.92 ± 0.27, respectively. There were no statistically significant differences in image quality, image noise and lesion conspicuity between G300 and G600 s (*P* > 0.05). The differences in lesion quality, image noise and lesion conspicuity between G150 and G300 were not statistically significant (*P* = 0.164, 0.255 and 0.112, respectively). There were also no statistically significant differences in lesion conspicuity between G150 and G600 s (*P* = 0.229). All images in G150 s were rated between 3 and 5, and the scores of lesion conspicuity in all groups were greater than 3. The overall quality of G60 s was 3.21 ± 0.46, and the image noise was 2.73 ± 0.59.

**Fig. 2:**
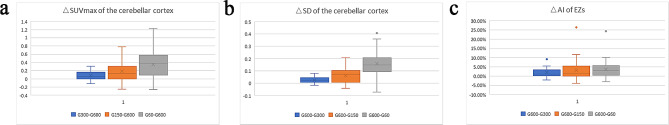
Box plots showing the change of ipsilateral cerebellar SUVmax, SD, and AI of EZs. The change of each index was calculated as the difference between G300 s, G150 s and G60 s of each patient and G600 s, respectively. (**a**) The SUVmax in the ipsilateral cerebellar cortex gradually increased as acquisition time decreased. G60 s-G600 s and G150 s-G600 s were significantly higher than G300 s-G600 s (*P* < 0.05). (**b**) The uptake of SD in the ipsilateral cerebellar cortex significantly increased as acquisition time decreased (*P* < 0.05). (**c**) There was no significant difference in AI difference between the groups with shortened acquisition time. The differences between G300 s-G600 s and G150 s-G600 s, G300 s-G600 s and G60 s-G600 s and G150 s-G600 s and G60 s-G600 s were not significant (*P* < 0.05 and Bonferroni correction). The difference in AI between the two time points was marked as ∆AI

Since most epilepsy is not associated with ipsilateral cerebellar metabolism, the ipsilateral cerebellar gray matter or cerebellum is often recognized as normal brain tissue and used as a reference area clinically. We objectively evaluated the image quality by delineating ROIs in a homogeneous area of the ipsilateral cerebellar cortex referring to the liver, including SUVmax and SD. The SUVmean in the center area of EZs and the corresponding contralateral regions were measured and AIs were calculated, with G600 s serving as reference (Table 3). The changes from G600 s to G300 s, G150 s, G60 s were also evaluated (Fig. 2).

**Table 3:** SUVmax and SD of the ipsilateral cerebellar cortex and AI

	Group 600 s	Group 300 s	Group 150 s	Group 60 s
Cerebellar cortex SUVmax	4.91 ± 1.97	4.99 ± 1.97	5.09 ± 2.10	5.26 ± 2.05
Change of cerebellar cortex SUVmax†		0.08 ± 0.12	0.18 ± 0.27	0.35 ± 0.38
Cerebellar corte × SD	0.27 ± 0.14	0.30 ± 0.15	0.33 ± 0.16	0.43 ± 0.17
Change of cerebellar corte × SD†		0.03 ± 0.02	0.06 ± 0.06	0.16 ± 0.10
AI	53.02% ± 0.32	51.03% ± 0.31	49.84% ± 0.32	49.28% ± 0.33
Change of AI†		1.98% ± 0.03	3.18% ± 0.06	3.74% ± 0.05

All data were presented as mean ± SD

† The change of SUVmax, SD and AI between the different groups were presented as mean ± SD, using that of group 600 s as references

As the acquisition time was reduced, the change in SUVmax and SD of the ipsilateral cerebellar cortex increased gradually. The ∆SUVmax of the cerebellar cortex in G150 s-G600 s, and G60 s-G600 s was significantly higher than that of the G300 s-G600 s (*P* < 0.05).

### Epileptogenic zones detectability

**Fig. 3:**
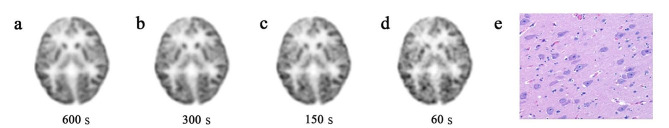
^18^F-FDG PET image of a 3-year-old female patient weighting 12.8 kg with a diagnosis of focal cortical dysplasia (FCD) by biopsy was reconstructed into brain axial views of 600, 300, 150 and 60 s (**a**–**d**). As the acquisition time was reduced to 60 s, the lesion was still identifiable. (**e**) Histopathology finding of the patient, characterized by architectural dysplasia and dysmorphic neurons

**Fig. 4:**
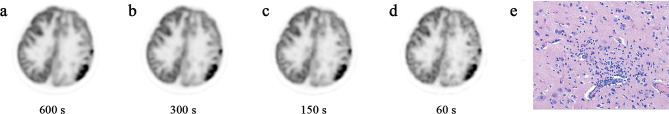
A 5-year-old man diagnosed with FCD IIId with Rasmussen encephalitis by pathology. The images showed lesions in left cerebral hemisphere for the group 60–600 s (**a**–**d**). Diffuse hypometabolism and focal hypermetabolism were observed in the left cerebral hemisphere, and the lesion detectability was visible from 600 s to 60 s. (**e**) Pathological image showed neuron degeneration and necrosis with astrocytic hyperplasia

Twenty-six EZs were found in 25 pediatric epileptic patients, including one patient with two EZs. Of these lesions, there are 11 were diffuse lesions in one hemisphere and 15 were focal lesions. Of the focal lesions, 9 were located in the temporal lobe, 4 in the frontal lobe, one in the parietal lobe and one in the occipital lobe. In G300 s, G150 s, and G60 s, all epileptogenic zones were identified by both radiologists, with a lesion detection rate of 100% relative to G600 s. Examples of serial PET images of focal and diffuse epileptic lesions are presented in Figs. 3 and 4, respectively.

## Discussion

Previous studies have revealed that PET is an important functional imaging technique for preoperative assessment of epileptic foci in epileptic patients, particularly for those with MRI-negative focal epileptic patients [5–8, 20]. The International consensus on the use of ^18^FFDG PET/CT in pediatric patients affected by epilepsy recommends a scan acquisition time of 20-min [3]. However, the sensitivity of the uEXPLORER has been increased approximately 4–5 times for imaging the brain [17]. Theoretically, increased sensitivity could lead to reduced scanning time in clinical applications [21, 22]. This could help to reduce motion artifacts, increase patient comfort and decrease the need for sedation [23]. Previous studies have shown that ultra-short acquisition time imaging is feasible in adult and pediatric cancer patients [16, 18]. Our study aimed to investigate the feasibility of short acquisition time in children with epilepsy.

This study investigated image quality and the ability to detect epileptogenic zones of time-dependent PET images on a TB PET/CT scanner with 194-cm-long axial FOV in children with epilepsy. In this proof-of-concept study, our results from the generated short acquisition time images showed that G300 s ensured both the optimal image quality (≥ 4) and EZs detective satisfaction referring to G600 s. The acquisition time of G150 s could result in sufficient EZs diagnostic performance, which had no statistical difference in lesion significance compared with G600 s and all images in G150 s were rated ≥ 3 on subjective PET image quality. Despite the image noise score in G60 s was less than 3 (2.73 ± 0.59), the scores of overall impression of image quality and lesion conspicuity in G60s were both greater 3 (overall quality, 3.21 ± 0.46; lesion conspicuity, 4.08 ± 0.74), which could meet the diagnostic requirements. Furthermore, AIs of all groups in this study were greater than 15% and the detection rate of EZs was 100%, although the overall image quality decreased with shortening of acquisition time significantly. Data in this study demonstrated that a short-time (150 s) PET was recommended for a clinical routine protocol. However, 60s PET scan was acceptable, especially for epileptic patients with probability of motion, claustrophobia or concerns that reduced the usage of sedation.

Since the ipsilateral cerebellar cortex had a relatively stable FDG metabolism, it was often recognized as normal brain tissue and used for FDG normalization [24]. The objective results showed that both the SD and SUVmax in the ipsilateral cerebellar cortex increased gradually as the scan time decreased. In PET images, image noise is assessed by SD of the SUV value, which means the extent of the SUV value deviates from the average SUV value in a ROI. In other words, the greater the SD, the greater the image noise. As the acquisition time decreased, the image noise increased and the image quality decreased gradually.

In our study, we subjectively and objectively evaluated the overall image quality and conspicuity of epileptic lesions. In this retrospective study, 11 diffuse lesions and 15 focal lesions were found and the lesion detection rate was 100% in all groups. However, the detection rate of lesions is closely related to the size, shape, metabolism and the surrounding environment of lesions, as well as the reader’s experience. A previous study showed that the diagnostic accuracy of seizure focus with PET/CT was approximately 78%, though the number of patients included in this study was small. Therefore, further multi-center study may be needed to investigate the detection rate of various epilepsy foci on TB PET/CT [25].

We concluded that sufficient subjective image quality and lesion significance could be maintained over a short acquisition time of 150 s, which was similar to the 180 s acquisition time that had been previously proven to be feasible with sufficient diagnostic satisfaction for pediatric epilepsy patients by Nicholas A. Shkumat et al. [4]. Research in low-dose pediatric TB ^18^F-FDG PET/CT scan by restructuring PET data to reduce count density showed that a reduced injected dose down to 1/10-dose (0.37MBq/kg) could be achieved with optimal image quality and sufficient lesion conspicuity of micro-lesions [16]. Our study has demonstrated that the scanning time of 150 s was sufficient to obtain diagnostic images of pediatric epileptic patients and that a 60 s PET scan could also have acceptable lesion detectability. Therefore, based on our data, reduction of administered activity in pediatric patients with epilepsy may be potentially achievable in future clinical practice.

This study has several potential limitations. Firstly, the sample size is small, thus the results may be subject to bias. Additionally, the study is limited to TB-PET/CT (uEXPLORER) and further exploration of minimal acquisition time with other PET machines is necessary. Lastly, while short acquisition time PET images were obtained by reconstructing low-count density images from 600 s PET data, this was not the actual short-time scanning. Nevertheless, our results may provide support for actual short-time TB PET/CT scanning for pediatric epilepsy patients in clinical work.

## Conclusions

Our study demonstrated that TB PET/CT with a 194-cm-long axial FOV can significantly shorten acquisition time while preserving image quality and diagnostic conspicuity of EZs in pediatric patients. Subjective image quality scores and objective image quality analysis revealed that a rapid PET/CT scan of 60 s was sufficient to detect lesions, particularly in patients who are unable to cooperate with extended PET scanning. However, a scan time of 150 s was found to be adequate to achieve satisfactory image quality in children with epilepsy.

## Data Availability

The datasets used and analysed during the current study are available from the corresponding author on reasonable request.
